# The Largest Systematic Review of Left Atrial Appendage Aneurysms: A Comprehensive Analysis of 216 Cases

**DOI:** 10.31083/RCM45129

**Published:** 2025-12-23

**Authors:** Klevis Mihali, Timo Mausinbaev, Julian Kreutz, Giulia Pasqualin, Massimo Chessa, Kevin Patrick Walsh, Colin Joseph Mcmahon, Pier Paolo Bassareo

**Affiliations:** ^1^Department of Cardiology, Angiology, and Intensive Care Medicine, University Hospital, Philipps-University of Marburg, 35037 Marburg, Germany; ^2^Adult Congenital Heart Disease (ACHD) Unit, IRCCS Policlinico San Donato, 20097 San Donato Milanese, Milan, Italy; ^3^Department of Cardiology, Vita-Salute San Raffaele University, 20132 Milan, Milan, Italy; ^4^School of Medicine, University College of Dublin, D04 C1P1 Dublin, Ireland; ^5^Department of Cardiology, Mater Misericordiae University Hospital, D07 R2WY Dublin, Ireland; ^6^Department of Cardiology, Children’s Health Ireland at Crumlin, D12 N512 Dublin, Ireland

**Keywords:** left atrial appendage aneurysm, echocardiography, cardiac imaging, cardiac surgery, arrhythmia, thromboembolism

## Abstract

**Background:**

Left atrial appendage aneurysm (LAAA) is a rare cardiac abnormality associated with thromboembolic events and arrhythmias. This systematic review aimed to provide a comprehensive evaluation of literature reports on the demographics, clinical presentation, electrocardiographic and imaging findings, treatment, and outcomes of patients with LAAA.

**Methods:**

A literature search was conducted using the PubMed, MEDLINE, and Scopus databases through September 2025. Only case reports and series explicitly describing LAAA were included. Extracted data included age, sex, clinical symptoms, electrocardiogram (ECG) characteristics, imaging findings, associated cardiac abnormalities, treatment modalities, and outcomes.

**Results:**

A total of 216 cases were included. The mean age at diagnosis was 30.41 ± 22.39 years, with a slight predominance of males (50.5%). Symptoms included palpitations (32.4%), dyspnoea (17.2%), and thromboembolic events (7.8%). Atrial fibrillation and flutter were the most commonly detected arrhythmias. Echocardiography was the most frequently used initial diagnostic tool, with computed tomography (CT) and magnetic resonance imaging (MRI) providing additional anatomical details. Chest X-rays often yielded non-specific findings. The mean aneurysm diameter was 6.87 ± 2.64 cm. Surgical treatment, mainly aneurysm resection, was the most commonly used approach (72.7%), while conservative and device-based therapies were applied selectively. Concomitant cardiac anomalies were present in 13.7% of cases and influenced case management. The mortality rate was 4.6%, although significant morbidity was observed. Multivariate logistic regression analysis revealed that atrial fibrillation/flutter was the sole variable significantly linked with clot formation/embolism (*p* < 0.05).

**Conclusion:**

LAAA is a rare, although clinically significant, entity with variable presentation and management challenges. However, early recognition and individualized treatment are essential. Further research is needed to define standardized diagnostic criteria and treatment guidelines.

## 1. Introduction

Left atrial appendage aneurysm (LAAA) is an exceptionally rare cardiac anomaly involving abnormal dilatation or outpouching of the left atrial appendage. First described in 1960 by Dimond *et al*. [1] as the “giant dog ear”, LAAA has since been reported sporadically in medical literature, primarily in individual case reports and small case series. It is considered both a congenital and, less commonly, acquired malformation that occurs across a wide range of age groups—from neonates to the elderly [2].

The precise pathophysiological mechanisms underlying LAAA are still not fully understood. Congenital forms are thought to result from localized muscular dysplasia or incomplete muscularization of the embryonic left atrium, leading to wall weakness in the appendage. Acquired LAAA may develop secondary to chronically elevated left atrial pressure, commonly in the context of mitral valve disease or left ventricular dysfunction [3].

**Fig. 1.**
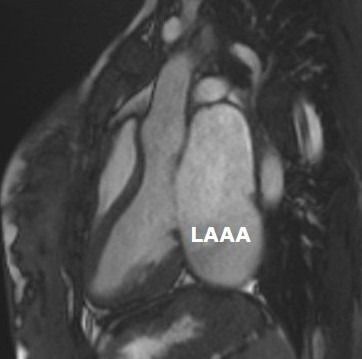
**Cardiac MRI**. Sagittal 3-chamber view from cardiac MRI showing a giant left atrial appendage dilatation. LAAA, Left atrial appendage aneurysm. MRI, Magnetic Resonance Imaging.

Clinically, LAAA may be asymptomatic and discovered incidentally during imaging, or present with a range of symptoms, including palpitations, dyspnea, chest pain, or systemic thromboembolic events such as stroke. The aneurysmal appendage may serve as a substrate for atrial arrhythmias and contribute to the onset of cardioembolic complications due to thrombus formation [4]. See Fig. 1 (Cardiac magnetic resonance imaging showing a dilated left atrium and LAAA as evaluated from a vertical 3-chamber long-axis view. Despite the size of the aneurysm, it contains no clot formation. This imaging technique is particularly useful in assessing the precise anatomical relationship of the aneurysm with surrounding structures, thus facilitating pre-surgical planning).

In view of its rarity, variable clinical presentation, and potential for serious complications, LAAA poses numerous diagnostic and therapeutic challenges. The present study aims to provide the most comprehensive and up-to-date systematic review of reported cases of LAAA, focusing on demographic features, clinical presentation, imaging findings, treatment strategies, and outcomes.

## 2. Search Methodology and Data Collection

A comprehensive literature search was conducted to identify all published cases of LAAA through September 2025. A search was undertaken on PubMed, MEDLINE, and Scopus databases using a combination of keywords and medical subject headings (MeSH), such as “left atrial appendage aneurysm”, “giant left atrial appendage”, “mitral valve disease”, and “rheumatic heart disease”. Boolean operators were employed to optimize sensitivity. Two authors (KM and PPB) extracted the intended data separately, and any disputes were discussed and resolved by a third investigator (MC). Reference lists of relevant articles were also screened manually to identify additional eligible studies.

**Fig. 2.**
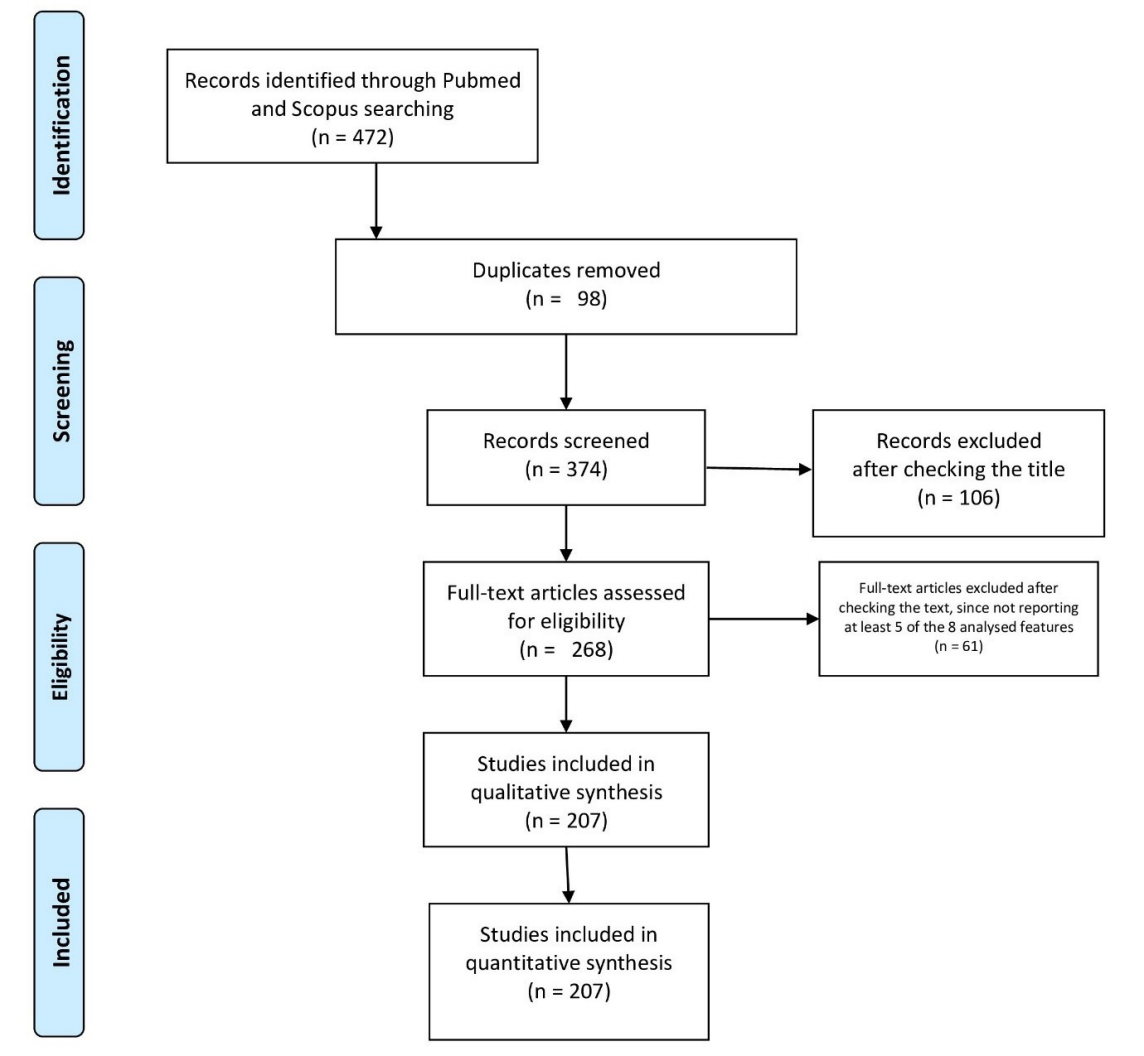
**PRISMA flow diagram**.

The inclusion criteria were extended to all case reports and case series explicitly documenting LAAA in human subjects. No observational studies were detected, and no articles were excluded based on language, publication date, or geographic origin. Manuscripts not reporting at least five of the eight analyzed features (age, sex, symptoms, electrocardiographic characteristics, imaging, association with other congenital cardiac abnormalities, treatment modalities, and outcome) were excluded. Cases referring solely to left atrial aneurysms not arising from the appendage were also excluded. This review adhered to the PRISMA (Preferred Reporting Items for Systematic Reviews and Meta-Analyses) statement [5]. See Fig. 2.

Four hundred and seventy-two single papers were initially selected, 265 of which were subsequently excluded (i.e., 98 were duplicates, 106 records were excluded after a title check, and 61 following an abstract check). At the end of the selection process, 207 studies were included for quantitative analysis (202 single case reports and 5 case series, the largest of which had studied 5 patients). Data were extracted using a standardized data collection form. Extracted variables included patient age, sex, clinical presentation, electrocardiographic features, chest X-ray findings, echocardiographic details, Computed Tomography (CT) and/or Magnetic Resonance Imaging (MRI) results, aneurysm dimensions, presence of associated cardiac abnormalities, treatment mode (surgical, conservative, or device-based), and clinical outcomes. Descriptive statistics, including frequency, percentages, mean values, and standard deviations for continuous variables, were calculated. The final dataset comprised a total of 216 individual cases of LAAA suitable for inclusion in the analysis [6, 7, 8, 9, 10, 11, 12, 13, 14, 15, 16, 17, 18, 19, 20, 21, 22, 23, 24, 25, 26, 27, 28, 29, 30, 31, 32, 33, 34, 35, 36, 37, 38, 39, 40, 41, 42, 43, 44, 45, 46, 47, 48, 49, 50, 51, 52, 53, 54, 55, 56, 57, 58, 59, 60, 61, 62, 63, 64, 65, 66, 67, 68, 69, 70, 71, 72, 73, 74, 75, 76, 77, 78, 79, 80, 81, 82, 83, 84, 85, 86, 87, 88, 89, 90, 91, 92, 93, 94, 95, 96, 97, 98, 99, 100, 101, 102, 103, 104, 105, 106, 107, 108, 109, 110, 111, 112, 113, 114, 115, 116, 117, 118, 119, 120, 121, 122, 123, 124, 125, 126, 127, 128, 129, 130, 131, 132, 133, 134, 135, 136, 137, 138, 139, 140, 141, 142, 143, 144, 145, 146, 147, 148, 149, 150, 151, 152, 153, 154, 155, 156, 157, 158, 159, 160, 161, 162, 163, 164, 165, 166, 167, 168, 169, 170, 171, 172, 173, 174, 175, 176, 177, 178, 179, 180, 181, 182, 183, 184, 185, 186, 187, 188, 189, 190, 191, 192, 193, 194, 195, 196, 197, 198, 199, 200, 201, 202, 203, 204, 205, 206, 207, 208, 209, 210, 211, 212]. See **Supplementary Table 1**.

### Statistical Analysis

Binary logistic regression analysis was used to identify predictors of the formation and embolism of LAAA-related clots (in the left atrium or LAAA), i.e., the odds ratios. Statistical significance was set at *p* < 0.05. Statistical power for multivariate logistic regression was greater than 0.80, ensuring a robust analysis. Power was calculated using established equations, where effect size (logarithm of the odds ratio) and standard error were derived from sample size and variance of predictor variables. Variables included age, sex, LAAA size, and atrial fibrillation/flutter. All the selected variables are known to be potential triggers of clot formation and were extrapolated from the reviewed case reports.

## 3. Results

A total of 216 cases were included in the final analysis. The main findings are summarized in Table 1.

**Table 1.** **Features of patients with left atrial appendage aneurysm**.

Male-to-female ratio	1.02/1
Mean age at diagnosis	30.41 ± 22.39 years
Symptoms	asymptomatic (42.6%, n = 92)
	palpitations (32.4%, n = 70)
	dyspnoea (17.2% n = 37)
	thromboembolic events (7.8%, n = 17)
ECG changes	Atrial fibrillation (33.3%, n = 72)
	Atrial flutter (8.1%, n = 17)
Diagnosis	by means of echocardiography (99.1%, n = 214)
	by means of cardiac magnetic resonance/computed tomography (73.1%, n = 158)
Mean dimensions of aneurysm	6.87 ± 2.64 cm
Associated congenital heart disease	13.7% (n = 31)
Death	4.6% (n = 10)

ECG, electrocardiogram.

Gender distribution was relatively balanced, with a modest predominance of males (50.5%). Age at diagnosis varied from infancy to advanced age, with a mean age at diagnosis of 30.41 ± 22.39 years. Common clinical symptoms included palpitations, shortness of breath, and thromboembolic events. The main electrocardiographic findings were atrial fibrillation and flutter. Echocardiography was the most frequently utilized initial diagnostic tool, with CT and cardiac MRI playing crucial roles in confirming diagnoses and clarifying anatomical details. Surgical interventions, mainly aneurysm resection or clipping, represented the most common therapeutic strategies, whereas conservative management and device-based therapies were less frequently applied. 13.7% of cases presented with concomitant cardiac anomalies that influenced therapeutic decisions and clinical outcomes. While mortality rates were relatively low, the morbidity associated with LAAA was significant. This underlines the importance of incidental imaging findings in at-risk populations and supports the case for increased awareness among non-cardiology specialists.

### 3.1 Clinical Presentation

Clinical symptoms were reported in the majority of cases. Palpitations were the most common symptom (32.4%), followed by dyspnea (17.2%) and thromboembolic events (7.8%), including cerebrovascular accidents. Additional symptoms included chest pain, fatigue, and presyncope. Notably, a significant portion of patients (27%) was asymptomatic and diagnosed incidentally during imaging performed for unrelated reasons.

### 3.2 Electrocardiographic and Imaging Findings

Electrocardiographic data were available for 186 patients (86.1%), indicating the presence of atrial fibrillation in 31.4% of cases and atrial flutter in 8.1%. Other arrhythmias and conduction abnormalities were less commonly observed.

Transthoracic and/or transesophageal echocardiography represented the primary diagnostic tool and was performed in 214 cases (99.1%). CT and cardiac MRI were used in 158 patients (73.1%) to confirm diagnosis and provide a detailed anatomical assessment. However, even the most advanced techniques at times fail to detect small or atypically located aneurysms, therefore highlighting the importance of maintaining a high index of clinical suspicion and adopting a multimodal diagnostic approach. Chest X-rays, performed in 73.3% of patients, frequently yielded non-specific findings, but occasionally revealed left-sided cardiomegaly.

### 3.3 Aneurysm Characteristics and Associated Findings

Mean dimension of aneurysms was 6.87 ± 2.64 cm. Thirty-one patients (14.3%) presented with associated cardiac anomalies, mainly atrial septal defects and mitral valve abnormalities.

### 3.4 Management and Outcomes

Surgical intervention was the first-line treatment strategy adopted in 157 patients (72.7%). Surgical techniques included aneurysmectomy, clipping, and concomitant correction of structural defects, when present. Conservative management was opted for in 42 cases (19.4%), typically in asymptomatic patients or those deemed high-risk surgical candidates. Device-based closure was reported in 13 cases (6.0%). In four cases, the therapeutic approach was not documented in the original publication, reflecting limitations in source reporting rather than incomplete data extraction.

Mortality directly attributable to LAAA was reported in 10 patients (4.6%). Postoperative outcomes were generally favorable, with resolution of symptoms and arrhythmias in the majority of surgically treated cases.

Long-term follow-up data were available for 104 of the 216 cases (48.1%), with a mean follow-up duration of 464.77 ± 89.6. days. The majority of patients remained asymptomatic during follow-up; however, detailed clinical outcomes were inconsistently reported and often limited in scope.

Statistics revealed how atrial fibrillation/flutter was the sole variable significantly linked with clot formation/embolism (*p* < 0.05) (Table 2).

**Table 2.** **Binary logistic regression analysis to identify predictors of thrombus/thromboembolism in the study population**.

95% CI of the Odds ratio Predictor	Odds ratio	Lower level	Upper level	*p* value
Age	0.98	0.96	1.00	0.79
Sex	0.89	0.35	2.33	0.89
Presence of atrial fibrillation/flutter	3.1	1.6	7.88	0.04
Size of aneurysm	0.96	0.84	1.08	0.88

CI, Confidence Interval. Significance was set at *p* < 0.05.

Regarding age, sex, and size of aneurysm, the related odds ratios (i.e., a number that quantifies the strength of the association between two events) values were less than 1. It means that their presence reduces the odds of the other event (thrombus/thromboembolism) occurring. Conversely, as to atrial fibrillation/flutter, the odds ratio value was greater than 1, which is associated with the risk of clot formation and migration.

No statistically significant differences in terms of LAAA size were detected between patients in atrial fibrillation or flutter and those in sinus rhythm (*p* = ns).

## 4. Discussion

This systematic review, currently the largest in the field, provides a fully comprehensive evaluation of LAAA to date, accounting for 216 cases reported across the literature. Although rare, LAAA represents a clinically consequential anomaly featuring a series of different manifestations, which is often diagnosed late and occasionally in the context of significant complications [6]. This analysis provides clarity on its epidemiological distribution, clinical manifestations, diagnostic modalities, therapeutic strategies, and associated outcomes.

LAAA has been observed across a wide age spectrum, with a mean age at diagnosis of approximately 30 years, aligning closely with earlier reports. In the same way as right atrial appendage aneurysms—featuring a strong male predominance—our cohort was likewise characterized by a slight male majority. This observation, consistent with findings from a recent 2024 review, may suggest the presence of shared embryological mechanisms underlying the development of atrial appendage [3, 210].

Clinical presentation remains heterogeneous. Palpitations and dyspnea were the most frequently reported symptoms, although more than one-quarter of patients were asymptomatic at diagnosis. This aligns with the findings of previous systematic analyses and underscores the diagnostic ambiguity that often surrounds LAAA [2, 3]. Notably, nearly 8% of cases presented with cerebrovascular events, reaffirming the role of aneurysm as a potential substrate for thromboembolism. In this context, arrhythmogenicity appears central: atrial fibrillation was identified in one-third of patients, supporting its mechanistic link to both embolic and hemodynamic consequences, as confirmed at multivariate analysis [95].

Imaging strategies were largely consistent with current practice. Transthoracic and transesophageal echocardiography were the main methodologies applied, in conjunction with advanced imaging techniques such as CT and MRI to provide added value in anatomic delineation and preoperative planning [107, 127]. Despite the use of these advanced technologies, diagnosis was often delayed, highlighting the need for increased clinical awareness, particularly in the presence of unexplained arrhythmias or embolic events.

Approximately 14% of patients presented with concomitant structural heart disease, the most common of which included atrial septal defects, patent ductus arteriosus, and mitral valve anomalies [50, 51, 58, 175]. While no causality should be inferred, the clustering of congenital anomalies suggests a potential developmental basis in select cases, and highlights the importance of a detailed cardiac workup in young patients with LAAA.

Importantly, aneurysm size was characterized by marked variability, with diameters ranging from approximately 1 cm to more than 15 cm. Our analysis yielded a mean aneurysm diameter of 6.87 ± 2.64 cm across 176 quantifiable cases. This considerable anatomical diversity likely influences both clinical presentation and therapeutic decision-making. While smaller aneurysms may remain clinically silent, larger or thrombus-filled aneurysms often require surgical management to mitigate risks of embolization or rupture [163].

Treatment strategies were largely dictated by symptom burden, aneurysm dimensions, and thromboembolic risk. Surgical resection or clipping represented the main approach and was associated with excellent outcomes [14]. In appropriately selected patients, particularly asymptomatic patients or those at high surgical risk, conservative management or percutaneous occlusion was successfully implemented. Device-based LAAA closure, although reported in only 6% of cases, represents a promising alternative in anatomically suitable patients [27, 53]. In the examined case reports, no complications associated with device implantation were reported, despite the challenges represented by the large size of the left atrial appendage. In patients with non-valvular atrial fibrillation, the left atrial appendage represented the source of clot development in 91–99% of cases. Accordingly, oral anticoagulation treatment for stroke prevention has become the standard of care in these patients. Nevertheless, oral anticoagulants are associated with a risk of bleeding, and their efficacy depends on optimal patient compliance [213]. Generally speaking, the efficacy of device implantation, first introduced more than 20 years ago, is deemed on a par with oral anticoagulants, although, conversely to medical treatment, it is not associated with long-term bleeding. Following significant improvements in procedural safety over the years, left atrial appendage closure, largely achieved using a catheter-based, device implantation approach, is increasingly applied in the prevention of thromboembolic events in patients unable to achieve effective anticoagulation [214]. However, in an LAAA setting, specific criteria relating to the choice of the most appropriate candidates for device closure are still lacking [215].

The prognosis for LAAA, when appropriately managed, is generally favorable. In our cohort, the mean follow-up duration across the 104 documented cases was approximately 465 days, although featuring a high degree of variability (± 89.6 days). The majority of surgically treated patients achieved resolution of their symptoms and arrhythmias, supporting the efficacy of operative intervention. However, the considerable heterogeneity and limited duration of follow-up data hamper the drawing of definitive conclusions with regard to long-term durability, recurrence risk, or late complications. Furthermore, treatment strategies were not reported in 2.4% of cases, highlighting the presence of persistent gaps in the literature.

Taken together, our findings highlight the critical need for earlier recognition and tailored management strategies for LAAA. A standardized diagnostic algorithm, including multimodal imaging, thromboembolic risk stratification, and structured follow-up, would result in improved clinical outcomes and a reduction in missed or delayed diagnoses. Despite the rarity of LAAA, taking into account the potential for serious complications, this abnormality should be considered in the differential diagnosis of arrhythmias and embolic events, particularly in younger patients.

The present study, however, features several important limitations. Firstly, it is based on a retrospective analysis of cases previously reported in the literature, which inherently introduces some degree of bias, namely selection and publication bias. Moreover, since asymptomatic cases of LAAA frequently go undetected, the cases included may not accurately reflect the broader population affected by LAAA. Another limitation is the lack of a universally accepted definition for what constitutes an LAAA. Foale and colleagues proposed criteria for use in diagnosing congenital left atrial aneurysms, to include the following: (1) origination from an otherwise normal atrial chamber, (2) a clearly defined connection with the atrial cavity, and (3) an intrapericardial location that distorts the left ventricle [216]. Despite this definition, by far the most comprehensive in the Authors’ opinion, an unequivocal agreement on the size threshold for classification of an aneurysm is still lacking. Measurement techniques likewise feature a wide variation, with some studies reporting the maximum width and depth obtained using transthoracic echocardiography (TTE), whilst others use two-axis measurements of the neck via transesophageal echocardiography (TEE), and some completely fail to specify the measurement method adopted. In an autopsy study of 500 normal hearts, Veinot *et al*. [217] provided baseline LAA sizes across different ages and sexes. We maintain that a standardized definition of LAAA is mandatory in order to facilitate more effective reporting and management of this entity. Our analysis was based on single case reports or very small case series, and it was impossible to ascertain whether or not the definition provided by Foale had been used. Indeed, the analysis we conducted adopted a multivariate logistic regression with a limited number of variables, thus yielding a somewhat limited statistical power and a high potential for bias due to the heterogeneity of the collected literature sources. It proved impossible to provide a quality score for the included studies due to the lack of extensive investigations in the field. Moreover, due to the nature of the included literature (mostly case reports and small case series), the level of evidence is limited, and follow-up data are lacking in approximately fifty percent of cases. Future work should focus on the development of multicenter registries and prospective studies for the purpose of establishing robust, evidence-based guidelines to be used in the diagnosis, surveillance, and treatment of this often-overlooked condition [218, 219].

Additionally, LAAA might be considered part of a wider and more modern concept, i.e., atrial cardiomyopathy. The latter is a term used to describe any structural, architectural, contractile, or electrophysiological change affecting the atria that has the potential to produce clinically relevant aftermaths. It is not a single disease, but rather a pathophysiological concept encompassing different atrial abnormalities, grouped as follows according to the EHRA (European Heart Rhythm Association) classification:

-Type I–Mostly cardiomyocyte-dependent changes. It is characterised by primary abnormalities of the atrial muscle cells. LAAA, often resulting from pectinate muscle dysplasia, might belong to this type [210].

-Type II–Mostly fibrotic changes. It is characterized by interstitial fibrosis and collagen deposition, leading to stiff atria, conduction slowing, and increased arrhythmia risk.

-Type III–Combined cardiomyocyte pathology and fibrosis. It is characterized by both myocyte damage and fibrosis.

-Type IV–Primarily non-collagen infiltration/deposits such as in amyloidosis, hemochromatosis, and fat infiltration [220].

LAAA might itself contribute to or be a manifestation of a broader atrial cardiomyopathy. The altered anatomy and poor contractility of the LAA in an aneurysm may result in sluggish blood flow, promoting clot formation and potentially triggering arrhythmias. However, whether LAAA is the reflection of a severe underlying atrial cardiomyopathy (as the association with heart failure and atrial fibrillation might suggest) or a primary disease due to embryological reasons and tissue weakness is still under debate.

As mentioned above, future work should focus, as a matter of priority, on the development of a multicenter registry geared to use a universally accepted definition of LAAA.

## Availability of Data and Materials

All datasets on which the conclusions of a manuscript depend are shared in the supplementary material section.

## Supplementary Material

Supplementary material associated with this article can be found, in the online version, at https://doi.org/10.31083/RCM45129.
