# Supplementary material for: The Largest Systematic Review of Left Atrial Appendage Aneurysms: A Comprehensive Analysis of 216 Cases
Source: Rev Cardiovasc Med. 2025 Dec 23;26(12):45129. doi: 10.31083/RCM45129 (PMC12781024; doi:10.31083/RCM45129)
Supplement: Supplementary file 1 [file 2153-8174-26-12-45129-s1.zip › Supplementary Table 1.docx]

| **Author** | **Patient´s Age at diagnosis (years)** | **Gender** | **Clinical Presentation** | **ECG** | **Chest X-Ray** | **Echocardiography** | **CT/MRI** | **Diameters** | **Associated cardiac abnormalities** | **Outcome** |
| --- | --- | --- | --- | --- | --- | --- | --- | --- | --- | --- |
| Lin J, et al. [6] | 62 | Male | Atrial fibrillation; NSTEMI and stroke despite anticoagulation | Atrial fibrillation | Not reported | Yes (TOE) | CT | 7.8 × 5 × 9 cm | None | Surgical exclusion with AtriClip, |
| Matsumoto K, et al [7] | 52 | Female | Atrial tachycardia unresponsive to ablation | Tachycardia with negative P, in I/aVL, positive in V1, bimodal in II | Not reported | Not used for diagnosis | CT | Not numerically reported – described as “giant” | None reported | Thoracoscopic LAA clipping |
| Wei HQ, et al.[8] | 36 | Female | Palpitations | Narrow QRS tachycardia with 2:1 AV conduction | Not reported | Yes (TTE) | CT | 86 × 67 mm | None reported | Unsuccessful radiofrequency ablation LAA resection via VATS |
| Ishii H, et al [9] | 19 | Male | Asymptomatic | Arrhythmia noted as newborn (details unknown) | Protrusion of the left third arch | Yes (TTE) | Yes (CT and MRI) A | 80 × 50 × 40 mm | None reported | LAA resection |
| Coraducci F, et al. [10] | 47 | Male | Palpitations and epigastric pain | Atrial fibrillation with high ventricular rate and LBBB | Not reported | Yes (TTE and TOE) | Yes (MRI) | 68 × 70 × 54 mm | None reported | Conservative management with anticoagulation, antiarrhythmics, and loop recorder |
| Abuzahra S, et al. [11] | 33 | Male | Recurrent palpitations and chest discomfort | Normal sinus rhythm on ECG; atrial flutter on Holter | Prominent left heart border | Yes (TTE) | Yes (CT) | 8.0 × 6.0 cm | None reported | LAAA resection via median sternotomy |
| Barocelli F, et al. [12] | 85 | Male | Palpitations | Atrial fibrillation | Not reported | Yes (TEE) | Yes (CT) | Ostium: 44 × 32 mm; Landing zone: 34 mm; Length: 49 mm | Thrombus in left atrial appendage; previous aortic valve replacement, mitral annuloplasty, coronary artery by pass graft | Successful custom-made LAmbre™ closure. |
| Chan Y-J, et al. [13] | 1.5 months | Female | Asymptomatic | Right axis deviation, absent R-wave progression | Extreme cardiomegaly (cardiothoracic ratio: 0.82) | Yes (TTE) | Yes (CT) | 41 × 47 mm | Dextroposition of heart | Successful aneurysmectomy |
| Oreto L, et al. [14] | 0 (fetal diagnosis at 26 weeks gestation, confirmed neonatally) | Male | Asymptomatic at birth; | Not reported | Not described | Yes (TTE) | Yes (CT) | Not numerically reported (described as “giant”) | Aneurysmal ductus arteriosus (closed spontaneously) | Surgical resection on day 5 postpartum |
| Norozi K, et al.[15] | 6 | Male | Asymptomatic | Normal sinus rhythm | Unusual contour along left heart border | Yes (TTE) | Yes (MRI) | 5.9 × 3.1 × 1.9 cm | None reported | Successful surgical resection |
| Aksu T, et al. [16] | 45 | Male | Palpitations | Atrial flutter | Not reported | Yes (TTE) | Yes (CT) | Not numerically specified (described as "giant") | None | Successful radiofrequency catheter ablation; surgery avoided due to high operative risk |
| Streb W, et al. [17] | 67 | Female | Palpitations ischemic stroke despite NOACs | Atrial fibrillation | Not reported | Yes (TOE) | Yes (CT) | TEE: 63 × 40 × 39 mm; CT: 4.59 × 4.66 × 4.9 cm | Thrombus in left atrial appendage | Successful transcatheter LAA closure with Amplatzer Amulet |
| Chowdhury NA, et al. [18] | 5 | Male | Palpitations, chest pain, dizziness | Atrial arrhythmia (details not specified) | Not mentioned | Yes (TTE) | Not mentioned | Not reported | Mitral valve prolapse | Surgical resection of aneurysm + mitral valve annuloplasty + maze therapy |
| Yan W, et al. [19] | 54 | Male | Recurrent chest tightness, dizziness, palpitations, exertional symptoms | Sinus rhythm, and left atrial enlargement om baseline ECG; Holter: atrial and ventricular tachycardia | Prominent bulge at left cardiac border | Yes (TOE) | Yes (CT) | 7.2 × 6.7 × 4.0 cm | Mild mitral regurgitation; left ventricular compression by LAAA | Successful surgical resection |
| DeBose-Scarlett A, et al. [20] | 17 | Female | Syncope | Normal sinus rhythm | Not reported | Yes (TTE and TOE) | Yes (MRI) | 7.1 × 7.6 × 5.7 cm | None reported | Successful minimally invasive resection via left mini-thoracotomy |
| Ku L, et al. [21] | 40 | Male | Intermittent palpitations and precordial discomfort | Sinus bradycardia | Prominent outpouching of left cardiac border | Yes (TOE) | Yes (CT and MRI) | 6.6 × 5.6 cm | Compression of banana-shaped left ventricle | conservative management with anticoagulation |
| Li R, et al [22] | 35 | Male | Acute massive cerebral infarction; | Atrial fibrillation and atrial tachycardia, ST-elevation (V2–V5) | Enlarged left heart border | Yes (TOE) | Yes CT confirmed LAAA compressing LV wall | TEE: 9.3 × 5.7 cm; CT: 9.3 × 6.4 × 3.8 cm | None reported | Surgical resection |
| Sauza-Sosa JC, et al. [23] | 52 | Female | Dyspnea after recent COVID-19 pneumonia | Not reported | Not mentioned | Not used for diagnosis | Yes (CT and MRI) | Not reported numerically (described as “giant”) | None reported | Managed conservatively with anticoagulation; surgical or device closure considered after treatment for pulmonary aspergillosis |
| Kissami I, et al [24] | 55 | Female | Dyspnea | First-degree AV block; paroxysmal atrial fibrillation with spontaneous recovery | Not specified | Yes (TOE) | Yes (CT) | TTE: 65 × 22 mm; MRI: 67 × 40 mm | Thickened mitral valve, mitral annular disjunction, hypertrophic left ventricular segments, dilated coronary artery (8 mm) | Medically managed with beta-blocker, anticoagulant, and diuretic; awaiting surgery |
| Liu T, et al. [25] | 50 | Female | Asymptomatic | Not specified | Not reported | Yes (TTE) | Yes (CT) | 9 × 6 × 7 cm | Cavernous haemangioma connected to LAA by 0.8 cm pedicle | Emergency thoracotomy and surgical removal |
| Rawtani S. et al [26] | 3 months | Male | Asymptomatic | Not reported | Not reported | Yes (TTE) | Yes (CT) | ~4 cm | Small perimembranousVSD with tricuspid tissue, LAAA compressing left lower pulmonary vein | Excision via median sternotomy |
| Kothandam S, et al [27] | 17 | Female | Recurrent ischemic strokes; seizures; abnormal behavior | Normal | Not reported | Yes (TOE) | Yes (CT) | 10.8 cm; ostium 20 mm | Thrombus in the left atrial appendage | First-ever catheter-based closure with VSD occluder; |
| Yanli Z, et al [28] | 75 | Male | Chest pain, palpitations | Atrial fibrillation | Not specified | Yes (TTE) | No | 94 × 67 mm; thrombus: 48 × 26 mm; neck: 24 mm | Severe mitral regurgitation | Surgery declined; managed conservatively with oral anticoagulants and annual echo follow-up |
| Pan S, et al. [29] | Diagnosed prenatally at 32 weeks gestation, operated at age 1 year | Male | Asymptomatic | Not reported | Not mentioned | Yes (TTE) | Yes (CT) | Echo at birth: 48 × 30 mm; Pre-op echo: 51 × 32 mm; CT: 46 × 42 × 44 mm; Surgical: 100 × 80 × 45 mm | Patent ductus arteriosus | Successful surgical resection + PDA ligation |
| Misugi E, et al. [30] | 6 | Male | Asymptomatic | Ventricular preexcitation (Wolff-Parkinson-White); supraventricular tachycardia | Not reported | Yes (TTE) | Yes (CT) | Not specified in mm; CT: 12.9 mL/m² | Left ventricular non-compaction; multiple accessory pathways | Successful radiofrequency ablation |
| Evangeliou AP, et al. [31] | 54 | Male | Asymptomatic | Sinus rhythm | Not specified | Yes (TOE) | Yes (CT and MRI) | 4.6 × 6.8 cm; neck 7.5 cm² | None reported | Conservative management with oral anticoagulation |
| Jiang B, et al. [32] | 44 | Female | Acute cerebral infarction; right-sided paralysis | Sinus rhythm | Prominent left cardiac border; cardiothoracic ratio 0.78 | Yes (TTE) | Yes (CT) | 18 × 10 × 8 cm | Thrombus in the left atrial appendage | Surgical resection |
| Yakut K, et al. [33] | 4 | Male | Asymptomatic; murmur on routine exam | Sinus rhythm | Double density at right heart border; enlarged left cardiac silhouette | Yes (TTE) | Yes (MRI) | Echo: 56 × 116 mm; MRI: 56.7 × 84.1 mm | Moderate-severe mitral valve regurgitation | Surgery |
| Li M, et al. [34] | 46 | Male | Palpitations | Atrial fibrillation , | Not reported | Yes (TTE) | Yes (CT) | 62 × 31 mm | atrial septal defect | Surgery declined; managed conservatively |
| Khanra D, et al. [35] | 45 | Female | PAlpitations | Paroxysmal atrial fibrillation | Cardiomegaly with tented left heart border | Yes (TTE) | No | Not numerically reported (described as “giant”) | Mild mitral valve regurgitation; thrombus | Patient declined surgery; treated with anticoagulation and beta-blocker; |
| Harland DR, et al. [36] | 43 | Female | Recurrent supraventricular tachycardia and chest pain | Supraventricular tachycardia and paroxysmal atrial fibrillation | Mild cardiomegaly | Yes (TTE and TOE) | Yes (CT) | CT: 7.0 × 6.9 × 5.9 cm; Echo: 9.1 × 6.7 cm | None reported | Surgical resection + maze procedure |
| Teng P, et al. [37] | 26 | Female | Asymptomatic | Sinus rhythm | Cardiomegaly with prominent left heart border | Yes (TTE and TOE) | Yes (CT) | Echo: 8.7 × 5.0 cm; CT: ~8 × 7 cm | None reported | On-pump aneurysmectomy |
| Combes S, et al. [38] | 55 | Female | Palpitations | Recurrent incessant atrial tachycardia, then paroxysmal atrial fibrillation | Not reported | Yes (TTE) | Yes (CT) | Not specified numerically; “giant LAA” | None reported | Hybrid electrophysiologic and thoracoscopic exclusion with AtriClip |
| Das R, et al. [39] | 54 | Female | Severe shortness of breath | Not specified | Not reported | Yes (TTE and TOE) | Yes (CT) | 14 × 11 × 8.5 cm | None reported | Surgical resection via limited thoracotomy |
| Aydin Sahin D, et al. [40] | 0 (newborn) | Female | Heart murmur | Sinus rhythm | Cardiomegaly, prominent left heart border | Yes (TTE) | Not performed | 35 × 26 mm | None reported | Surgery |
| Nezafati MH, et al. [41] | 26 | Male | Acute neurologic event with hemiparesis, aphasia, facial nerve palsy | Atrial fibrillation | Prominent convexity on left heart border | Yes (TTE) | Yes (CT) | Echo: 10 × 15 cm; Surgery: 15 × 12 × 8 cm; Thrombus: 8 × 8 cm | Thrombus | Surgical resection |
| Morin J, et al. [42] | 50 | Male | Dyspnea, orthopnea, paroxysmal nocturnal dyspnea | Supraventricular tachycardia | Mediastinal mass; pericardial calcifications | Yes | Yes (CT) | 8 cm | Suspected partial pericardial agenesis | Initial ablation failed; antiarrhythmic drugs to control symptoms; surgery deferred |
| Chen Y, et al. [43] | 36 | Female | Chest pain; i | Normal | Enlarged heart shadow on annual follow-up | Yes (TTE)² | Yes (CT) | Echo: 96 × 55 mm; Surgical: 100 × 80 × 45 mm | None reported | Surgical resection |
| Valentino MA, et al. [44] | 51 | Female | Palpitations | Atrial flutter | Mass-like density in left anterior mediastinum | Yes (TTE) | Yes (CT) | Max: 11.0 × 8.0 × 8.4 cm | Mitral regurgitation; mild pulmonary hypertension (later) | Managed conservatively for 5 years; resection after aneurysm enlargement |
| Hui C, et al. [45] | 19 | Female | Asymptomatic | Sinus rhythm, | Cardiothoracic ratio 0.6–0.7; spherical heart | Yes (TTE) | Yes (CT) | TTE: 135 × 100 × 80 mm; Surgical: 100 mm long | Compression of left ventricle | Surgical resection |
| Zhari B, et al. [46] | 14 | Male | Rapidly progressive dyspnea, palpitations, dizziness and fainting | Atrial fibrillation | Cardiomegaly with prominent left heart border | Yes (TTE) | Yes (CT) | 70 × 50 mm | None reported | Surgical resection |
| Bharati A, et al. [47] | 11 months | Male | Incidental finding during pneumonia evaluation | Not reported | Cardiomegaly with left heart border bulge | Yes (TTE) | Yes (MRI) | 7 cm | Compression of left ventricular basal wall and left superior pulmonary vein | Surgical resection |
| Wagdy K, et al. [48] | 16 | Male | Dyspnea and palpitations | Atrial tachycardia | Marked left atrial enlargement | Yes (TTE) | Yes (CT and MRI) | 8.0 × 7.0 cm | Compression of left anterior descending artery with mildly impaired left ventricular systolic function (EF 40%) | Surgical resection |
| Kahraman F, et al. [49] | 57 | Female | Palpitations and heart murmur | Atrial fibrillation and left ventricular hypertrophy | Not reported | Yes (TTE) | No | Not specified numerically | Hypertrophic cardiomyopathy; moderate to severe mitral regurgitation | Managed with anticoagulation after rhythm conversion to sinus |
| Pawar R, et al. [50] | 4 | Female | Cyanosis since infancy | Not detailed | Not mentioned | Yes (TTE) | No | Not specified numerically | Tricuspid atresia, pulmonary atresia, unrestrictive atrial and septal defects, patent ductus arteriosus, hypoplastic right ventricle, bilateral superior vena cava | Advised early surgery; patient lost to follow-up |
| Brazier A, et al. [51] | 24 | Female | Orthopnea, paroxysmal nocturnal dyspnea, stridor | Atrial fibrillation | Not reported | Yes (TTE) | Yes (MRI) | 8 cm | Severe mitral stenosis; mild aortic stenosis; Hurler-Scheie syndrome | Urgent mitral valve replacement with 17 mm prosthesis + LAAA resection |
| Ruttkay T, et al [52] | 23 | Female | Dyspnea and chest pain | Sinus rhythm | Widened mediastinum on left heart side | Yes (TTE and TOE) | Yes (MRI) | 6 × 4 cm; neck 2.5 cm | None reported | Totallendoscopic resection with stapler |
| Salido-Tahoces L, et al. [53] | Not specified (adult) | Not specified | Asymptomatic | Chronic atrial fibrillation | Not reported | Yes (TTE) | Yes (CT) | 26 × 32 mm neck 10 mm | None reported | Successful percutaneous closure with 34 mm AMULET devices |
| Kawano H, et al. [54] | 22 | Male | Asymptomatic | Normal ECG | Suspected mediastinal mass | Yes (TTE and TOE) | Yes (CT) | Not numerically reported | None reported | Managed conservatively |
| Zeng H, et al. [55] | 9 | Female | Cough | Not reported | Cardiomegaly | No | Yes (CT and MRI) | 8.9 × 10.2 × 10.1 cm | Severe mitral regurgitation; single papillary muscle | Surgical resection and mitral cleft repair |
| Gan GCH, et al. [56] | 45 | Female | Ischaemic chest pain; history of previous hemorrhagic stroke | Sinus rhythm | Prominent left heart border | Yes (TTE and TOE) | Yes (CT) | CT: 57 × 42 × 40 mm; Pathology: 67 × 37 × 22 mm | Large thrombus within aneurysm cavity | Surgical excision |
| Clark JB, et al. [57] | 23 | Female | Palpitations, dyspnea, fatigue | Atrial flutter | Enlarged cardiac silhouette with prominent left atrium | Yes (TTE and TOE) | Not used | 5.4 × 8.8 cm²; neck: 2.2 cm | None reported | Successfully resected via left thoracotomy |
| Yang EH, et al. [58] | 44 | Female | Heart failure symptoms | Right axis deviation, right ventricular hypertrophy | Enlarged cardiac silhouette; suspected mediastinal mass | Yes (TEE) | Yes (CT) | Length: 95 mm | Secundum atrial septal defect, pulmonic valve stenosis | Surgical closure of atrial septal defect and left atrial appendage resection |
| Oz A, et al. [59] | 3 | Male | Chronic cough | Normal sinus rhythm | Enlargement of left heart border | Yes (TEE) | Yes (MRI) | 4.2 × 3 × 3.5 cm | None reported | Surgical aneurysmectomy |
| Bouallouche SA, et a. [60] | 35 | Male | Dizziness; vertebrobasilar ischaemic strokes | Atrial fibrillation | Not specified | Yes (TTE and TOE) | Yes (MRI) | 11.2 × 4 cm | Thrombus inside aneurysm | Surgical resection |
| Youssef AA, et al. [61] | 22 | Not specified | Palpitations | Atrial fibrillation | Suspicious prominent cardiac silhouette | Yes (TTE) | Yes (MRI) | 9 × 7 cm | Significant compression of adjacent cardiac structures | Surgical resection |
| Kuiten WMM, et al. [62] | 39 | Male | Cerebellar infarction | Atrial fibrillation | Convex left upper cardiac contour | Yes (TTE and TOE) | Yes (CT and MRI) | 7 × 7 × 2.5 cm | None reported | Surgical resection |
|  | 69 | Male | Dyspnea, permanent afib | Atrial fibrillation | No | Yes ( TTE and TOE) | Yes (CT) | 7 × 5.5 × 2.5 cm | None | Surgical resection |
| Miljak T, et al. [63] | 69 | Male | Dyspnoea | Atrial fibrillation | Not done | Yes (TTE) | Yes (CT) | 7 × 5.5 × 2.5 cm | None | Surgical resection |
| Çakıcı M, et al. [64] | 62 | Male | Palpitations, heart failure | Atrial fibrillation | Not reported | Yes (TOE) | Not done | Not specified numerically (described as “giant”) | Heart failure; LVEF 30%; thrombus | Initial antiplatelet/heparin/tirofiban ineffective; thrombus resolved after 6 weeks of warfarin therapy |
| Clarke JR, et al. [65] | 0 (neonate) | Female | Asymptomatic at birth; hypotension on day 1 | Sinus tachycardia | Prominent bulge of the left heart border | Yes (TTE) | Yes (MRI) | Echo: 2.9 × 3.4 cm; MRI: 2.5 × 4.3 cm; intraoperative: 4 cm | Mild mitral regurgitation; compression and thinning of left ventricle; transient left ventriculardysfunction | Surgical resection |
| Di Salvo G, et al. [66] | 4 months | Male | Heart murmur | Not mentioned | Not mentioned | Yes (TTE) | Yes (CT) | Not numerically reported | Compression of left upper pulmonary vein | Successful surgical resection |
| Hassan M, et al. [67] | 10 | Female | Palpitations | Atrial fibrillation | Marked cardiomegaly | Yes (TTE) | Not done | 5.5 × 7.3 cm; neck: 3.5 cm | Left anterior descending artery compression; reduced left ventricular function; thrombus | Surgical resection with patch closure of neck |
| Sarin SS, et al.[68] | 2 | Male | Seizure followed by left-sided hemiparesis (cardioembolic stroke) | Sinus rhythm | Prominence of superior left heart border | Yes (TTE) | Yes (MRI) | 5.6 × 5.1 cm; neck: 1.3 cm | Thrombus | Surgical resection |
| Bhattarai A, et al. [69] | 3 months | Female | Asymptomatic | Sinus rhythm | Cardiomegaly with prominent left heart border | Yes – 2D echo: LAAA 45 × 60 mm with wide orifice | No CT/MRI; echo sufficient | 45 × 60 mm | Moderate mitral regurgitation; LV compression and displacement | Off-pump surgical resection; uneventful recovery; reduced MR; healthy at 1-year follow-up |
| Hof IE, et al. [70] | 42 | Male | Palpitations | Paroxysmal atrial fibrillation | Prominent left upper cardiac border; normal cardiothoracic ratio | Yes (TTE) | Yes (MRI) | 7 cm (length) | None reported | Successfully treated with ablation alone (no resection) |
| Gajjar T, et al. [71] | 29 | Male | Dyspnea and palpitations | Not specified | Abnormal bulge at left heart border | Not reported | Yes (CT) | 15 × 7 × 7 cm (surgical specimen) | None reported | Surgical resection |
| Parakh N, et al. [72] | 30 | Female | Palpitations and dyspnea | Atrial fibrillation, right axis deviation, left ventricular hypertrophy | Cardiomegaly with left-sided hump | Yes (TTE) | Not done | LA: 12 cm; LAA base: 5.6 cm | Severe rheumatic mitral valve disease with regurgitation, moderate tricuspid regurgitation, pulmonary hypertension | Surgical left atrial appendage ligation and mitral valve replacement |
| Knapp M, et al. [73] | 33 | Female | Nonspecific chest pain | Sinus rhythm | Normal | Yes (TTE and TOE) | Yes (CT) | 6 × 3 cm | None reported | Declined surgery; no medication started; under observation |
| Tietge W, et al. [74] | 42 | Male | Palpitations | Paroxysmal atrial fibrillation | Not reported | Yes (TTE) | Not done | Not specified numerically | None reported | Managed conservatively; no intervention performed |
| Zhang P-F, et al. [75] | 29 | Female | Exertional palpitations and shortness of breath | Sinus tachycardia | Not reported | Yes (TTE) | Not done | 22 × 15 × 15 cm; orifice: 3 cm | Left ventricular compression | Surgical resection |
| Bilge M, et al. [76] | 56 | Male | Epigastric pain under emotional stress | Sinus rhythm, pathological Q-waves (II, III, aVF) | Mildly prominent upper left atrial border | Yes (TTE and TOE) | Not done | 3.3 × 6.1 cm | Severe ischaemic mitral regurgitation | Percutaneous intervention failed; managed medically without surgery |
| Smeglin A, et al. [77] | 31 | Female | Lightheadedness, dizziness, intermittent palpitations | Atrial fibrillation/flutter | Abnormal upper left heart border | Yes (TTE and TOE) | Yes (CT and MRI) | 7.2 × 3.9 cm | Compression of left ventricular anterolateral wall | Surgical resection + Maze |
| Dumitrescu A, et al. [78] | 18 months | Female | Asymptomatic | Abnormal ECG (no further features have been reported)) | Marked cardiomegaly | Yes (TTE) | Yes (CT and MRI) | Not numerically reported | Shared wall with left ventricle; abnormal circumflex artery course | Initial plication (off-bypass) due to coronary course; patch closure later; residual growth observed but patient stable |
| Yong HS, et al. [79] | 67 | Female | Atypical chest discomfort; history of embolic stroke | Atrial fibrillation | Markedly prominent left cardiac border | Yes (TOE) | Yes (CT) | 4 × 6 × 8 cm | Thrombus inside aneurysm | Patient refused surgery; managed with oral anticoagulation |
| Crean AM, et al. [80] | Not stated (young adult) | Male | Breathlessness; dysmorphic facial features | Not specified | Not reported | Yes (TTE) | Yes (MRI) | Not quantified | Biventricular non-compaction | Not reported |
| Conradi G, et al. [81] | 42 | Male | Dyspnea, palpitations, chest pressure while supine | Paroxysmal atrial fibrillation | Large para-cardiac shadow (misinterpreted as pericardial effusion) | Tes (TTE) | Yes (MRI) | 93 × 47 × 69 mm | Mild mitral regurgitation; left ventricular compression; thrombus | Surgical resection + Maze |
| Moreno-Martínez FL, et al. [82] | Teenager (exact age not given) | Female | Embolic stroke | Atrial fibrillation | Not reported | Not explicitly mentioned (assumed yes) | Yes (CT) | Not specified | None reported | Successful surgical resection |
| Ulucam M, et al. [83] | 54 | Female | Palpitations, dyspnea, cerebral embolism (transient hemiparesis) | Paroxysmal atrial fibrillation | Prominent left cardiac border | Yes (TTE and TOE) | Yes (CT and MRI) | Echo: 11 cm long, 3 cm wide; surgical: 8 × 7.5 × 1 cm | Mural thrombi inside aneurysm | Surgical resection |
| Lekkerkerker JC, et al. [84] | 40 | Female | Asymptomatic | Sinus rhythm; | Prominent superior left heart border | Yes (TTE and TOE) | Yes (MRI) | 5.9 × 3.1 cm | None reported | Managed conservatively with oral anticoagulation and echo follow-up |
| Thomas E, et al. [85] | 4 | Male | Asymptomatic | Sinus rhythm with right bundle branch block | Borderline cardiomegaly with “shouldering” of the left cardiac border | Yes (TTE) | Yes (MRI) | Not specified numerically | Structurally normal heart | Managed conservatively |
| Kiaii B, et al. [86] | 35 | Male | Dyspnea, reduced exercise tolerance | Chronic atrial fibrillation | Enlarged left atrial contour | Yes (TOE) | Yes (MRI) | 90 × 40 mm; neck 20 mm | History of viral myocarditis 4 years prior; thrombus | Successful minimally invasive endoscopic resection + cryoablation |
| Kunishima T, et al. [87] | 25 | Male | Headache | Sinus bradycardia; biphasic/wide P waves in chest leads | Protrusion of the third arch; cardiothoracic ratio 50% | Yes (TTE and TOE) | Yes (MRI) | 4 × 3 × 2 cm (surgical) | Thrombus | Surgical resection |
| Rikitake K, et al. [88] | 45 | Male | Not specifically mentioned | Not reported | Mediastinal shift to right | Presumed – suggested by diagnosis | Not reported | 13 × 10 cm | Chronic massive mitral regurgitation | Mitral valve replacement with chordal sparing and left atrial plication via the aneurysmal left atrial appendage |
| Gold JP, et al. [89] | 34 | Female | Episodes of severe palpitations with shortness of breath | Sinus tachycardia | Significant prominence of superior left heart border | Yes (TOE) | Yes (CT and MRI) | 10 × 15 cm (intraoperative); pathology: 18 × 18 × 2 cm | None reported | Surgical resection |
| Frambach PJGM, et al [90] | 15 | Male | Sudden chest pain, palpitations, dizziness; diagnosed with myocardial infarction | Atrial fibrillation, negative T-waves in lateral leads | Cardiomegaly, enlarged left side | Yes (TTE) | Not done | Echo: 15 cm; Intraoperative: ~20 cm | Left ventricular compression; thrombus | Surgical aneurysmectomy |
| Lipkin D, et al. [91] | 18 | Female | Irregular pulse | Atrial fibrillation | Normal size heart with prominent left heart border | Yes (TTE) | Yes (CT) | Not numerically specified | None reported | Surgical excision via thoracotomy |
| Van der Hauwaert LG, et al. [92] | 2 | Female | History of bronchitis and wheezing; asymptomatic regarding heart | Normal ECG | Grossly distorted cardiac silhouette, rounded bulge left border | Yes (TTE) | Not done | 12 × 4 × 4 cm; 3 cm orifice to left atrium | Slight left ventricular compression | Surgical excision |
| Dimond EG, et al. [93] | 47 | Male | Headache and muscle cramps (incidental finding) | Inverted T waves (I, aVL); unique deflection before QRS | Wedge-shaped density over left heart border | No echo (pre-echo era) | Not done | 11.0 × 3.5 × 2.5 cm | Herniation through 2.5 cm pericardial defect | Surgical resection |
| Mittal A, et al. [94] | 28 | Male | Reduced exercise tolerance post-COVID | Atrial fibrillation and typical atrial flutter | Distorted left cardiac border | Yes (TTE and TOE) | Yes (CT and MRI) | Max: 8.4 × 5.5 cm; ostial 1.6 cm | Mild left ventricular dysfunction; compression of left ventricular wall | Surgical resection + Cox-Maze IV |
| Qin K, et al. [95] | 20 | Male | Asymptomatic | Normal ECG | Bulging upper segment of heart | Yes (TTE) | Yes (CT) | Intraoperative: 7 × 7 cm | Mild mitral regurgitation; compression of left upper pulmonary vein and pulmonary trunk | Surgical resection |
| Zhang H, et al. [96] | 61 | Female | Dyspnea and palpitations over 7 years; renal embolism | Atrial fibrillation | Prominent left heart border | Yes (TTE and TOE) | Yes (CT) | Max: 8.1 × 4.6 cm | Left atrial appendage with thrombus; renal artery embolism; mild left ventriculardysfunction | Surgical resection + Maze procedure |
| Ayala Torres JD, et al. [97] | 67 | Female | Dyspnea, functional decline | Not stated | Left cardiac mass detected | Yes (TTE) | Yes (CT) | 12 cm | Compression of left cardiac chambers; thrombus | Surgical resection; patient died of infectious complications (urinary sepsis by Klebsiella spp.) |
| Fadel R, et al. [98] | 78 | Male | Palpitations; history of intracranial hemorrhage | Persistent AF | Not reported | Yes (TTE) | Yes (CT) | Not reported | None reported | Successful left atrial appendage occlusion with WATCHMAN™ FLX device |
| Chraibi H, et al. [99] | 58 | Female | Asymptomatic | Atrial fibrillation | Not specified | Yes (TTE) | Yes (MRI) | 72 × 38 mm; neck 37 mm | Caseous mitral annular calcification; mitral annular disjunction; bi-leaflet ballooning | Surgery declined; managed with beta-blockers and oral anticoagulation |
| Bigdelu L, et al. [100] | 38 | Female | Mild dyspnea during pregnancy | Sinus rhythm | Not reported | Yes (TTE and TOE) | No CT/MRI due to pregnancy | 2.9 × 8 cm | Paravalvular mechanical mitral valve leak | Conservatively managed due to pregnancy |
| Atasayan V, et al. [101] | 19 days | Female | Hydrops fetalis | Sinus rhythm | No | Yes (TTE) | Yes (CT) | Not specified numerically | None | Surgical resection at 1 month; patient died post-op due to hypotension and multi-organ failure |
|  | 5 days | Male | Asymptomatic | Sinus rhythm | No | Yes (TTE) | Yes (CT) | 3.2 × 2.8 cm | None | Conservatively managed |
| Yamashita N, et al. [102] | 0 (newborn) | Male | Initially asymptomatic; later respiratory distress and feeding difficulties | Sinus rhythm with normal axis | Cardiomegaly with prominent left heart border | Yes (TTE) | Yes (DT and MRI) | Surgical: 65 × 35 × 30 mm | Compression of pulmonary artery; mild-moderate pulmonary stenosis | Surgical resection at 29 days old |
| Choi YJ, et al. [103] | 53 | Female | Dyspnea and mild palpitations | Not stated | Cardiomegaly; bulging upper left heart border | Yes (TTE) | Yes (CT and MRI) | 24 mm (orifice), 49 mm (width), 71 mm (length) | Small thrombus on histology | Successfully resected via video-assisted thoracoscopic surgery |
| Isa H, et al. [104] | 73 | Female | Cardiogenic stroke | No atrial fibrillation on 7 Holter exams (atrial fibrillation occurred during surgery) | Enlargement of left atrial appendage | Yes (TTE) | Yes (CT) | 5.5 × 4.0 × 1.5 cm | None reported | Surgical resection |
| Gray R, et al. [105] | 23 | Male | Intermittent exertional palpitations and presyncope | Sinus rhythm with broad, bifid P wave | Enlarged left heart border | Yes (TTE and TOE) | Yes (CT and MRI) | 75 × 41 mm; neck 19 mm | Indentation of basal-to-mid lateral left ventricular wall | Surgical clipping with Atriclip via mini-thoracotomy |
| Fnon NF, et al. [106] | 19 | Male | Sudden chest pain and collapse during vigorous physical exercise | Not specified | Not available | Not performed | Not performed | ~5 cm (autopsy) | Compression of left circumflex coronary artery; myocardial infarctions | Sudden cardiac death due to chronic myocardial ischemia from left atrial appendage compression; diagnosed post-mortem |
| Oyama S, et al. [107] | 80 | Male | Asymptomatic | Chronic atrial fibrillation | Not done | Likely used, but not mentioned | Likely used, but not detailed | Not reported | None | Surgical resection performed at 80; left atrial appendage found filled with thrombus; |
| Nakamura M, et al. [108] | 38 | Male | Palpitations and exertional dyspnea | Atrial fibrillation | Cardiomegaly | Yes (TTE and TOE) | Yes (MRI) | Not numerically stated | Left ventricular compression and deformation by aneurysm | Surgical clipping and resection of left atrial appendage + Maze procedure |
| Pradella M, et al. [109] | 38 | Male | Syncope, palpitations and atypical chest pain | Supraventricular tachycardia | Left-sided cardiomegaly | Yes (TTE) | Yes (MRI) | 5.7 × 5.3 × 8.2 cm | Moderate mitral regurgitation; left ventricular compression | Surgical resection |
| Sawicki KT, et al. [110] | 37 | Male | Intermittent palpitations, syncope | Sinus bradycardia; left atrial enlargement; paroxysmal supraventricular tachycardia | Prominent outpouching left cardiac border | Yes (TTE) | Yes (MRI) | Echo: 7.0 × 4.0 cm; CMR: 7.6 × 6.9 × 5.9 cm | Moderate mitral valve regurgitation | Surgical resection |
| Zalewska L, et al. [111] | 2 | Male | Asymptomatic | Normal sinus rhythm | Normal cardiothoracic index; left heart border bulge | Yes (TTE and TOE) | Yes (CT) | 50 × 38 × 49 mm | Left ventricular compression; | Surgical resection |
| Das N, et al. [112] | 3 | Female | Asymptomatic | Normal | Not specified | Yes (TTE) | Yes (CT) | Not numerically stated | Functionally bicuspid aortic valve | No intervention; stable at 1-year follow-up with no symptoms |
| Sasaki T, et al. [113] | Prenatal ( 0 years) | Female | Asymptomatic at birth | Sinus rhythm, no ST-T changes | Not specified | Yes (Foetal echo; postnatal TTE) | Yes (CT and MRI) | Echo: 30×18 mm; CT: huge | Mild left ventricular and left upper pulmonary vein compression; slight displacement of left circumflex artery | Surgical resection at 7 months |
| Rengifo LM, et al. [114] | 16 | Male | Dizziness, palpitations, shortness of breath | Sinus arrhythmia at rest; Holter: paroxysmal supraventricular tachycardia, atrial fibrillation/ flutter | Not specified | Yes (TTE) | Yes (CT) | CT: 63×50×75 mm | None | Thoracoscopic resection |
| Su X, et al. [115] | Prenatal (0 years) | Female | Asymptomatic | Sinus rhythm | Cardiomegaly at 12 months with left heart border prominence | Yes (Fetal echo and postnatal TTE) | Yes (CT) | CT: 46×42×44 mm; TTE up to 51×32 mm | Patent ductus arteriosus; left upper pulmonary vein and bronchial compression | Surgical resection and patent ductus arteriosus ligation at 12 months |
| Fan F, et al. [116] | 3 | Not specified | Referred for pneumonia | Not stated | Enlarged left cardiac silhouette | Yes (TTE) | Yes (MRI) | TTE: 4 × 4 cm; neck 2 cm | None reported | Surgical resection |
| Yoshihara S, et al. [117] | 48 | Male | Congestive heart failure | Atrial fibrillation | Convex bulge on left heart border | Yes (TTE) | Yes (CT) | 87 × 44 mm; ostium 35 × 27 mm; volume 69 mL | Thrombus in left atrial appendage; mild left ventricular dysfunction | Patient refused surgery; treated with oral warfarin; managed conservatively |
| Low ZK, et al. [118] | 0 (neonate) | Male | Respiratory distress at birth | Atrial flutter | Cardiomegaly | Yes (TTE) | Yes (CT) | 43 × 36 mm | Moderate-to-severe mitral regurgitation; thrombus in left atrial appendage | Surgical resection on day 11 of life; inadvertent left anterior descending artery injury repaired; uneventful recovery; mitral regurgitation improved |
| Belov DV, et al. [119] | 58 | Male | Dyspnea, dizziness on exertion, fatigue | Atypical atrial flutter | Convexity on left atrial border | (YES (TTE and TOE) | Yes (MRI) | 123 × 90 × 70 mm | Mitral insufficiency; left ventricular deformation; 60% left main coronary artery + circumflex stenosis | Surgical aneurysmectomy + mitral annuloplasty + left main coronary artery + circumflex bifurcation stenting; |
| Oda S, et al. [120] | 29 days | Male | Exertional dyspnea starting day 21 | Sinus rhythm | Not specified | Yes (foetal and postnatal TTE) | Tes (MRI) | 65 × 35 × 30 mm | Compression of left ventricle, right ventricular outflow tract, and pulmonary artery | Surgical resection |
| Yeung DF, et al. [121] | 22 | Male | Intermittent pleuritic chest pain and shortness of breath | Sinus bradycardia | 3.8 cm soft tissue density over left hilum (initially suspected mediastinal mass) | Yes (TTE and TOE) | Yes (CT and MRI) | 5.2 × 2.3 cm | None | Managed conservatively |
| Ergül Y, et al. [122] | 30 days | Male | Palpitatio | Broad QRS tachycardia, intermittent Wolff-Parkinson-White pattern | Not specified | Yes (TTE) | Not done | Not specified | Left ventricular dilatation, mitral regurgitation, patent foramen ovale | Successful radiofrequency ablation of accessory pathway; sinus rhythm restored |
| Tandon R, et al. [123] | 67 | Male | Class III exertional angina | Atrial flutter | Mass-like opacity in left anterior mediastinum | Yes (TTE and TOE) | Yes (CT) | 3D TTE: 5.74 × 5.24 × 5.18 cm; volume 76.3 mL | Severe alft anterior descending artery compression by left atrial appendage aneurysm; mild mitral regurgitation; thpmbus | Patient refused surgery; managed medically with anticoagulation and anti-anginal therapy |
| Wang B, et al. [124] | 46 | Male | Chest discomfort for 3 months | Sinus rhythm, notched P wave | Greatly enlarged left heart border | Yes (TTE and TOE) | Yes (CT and MRI) | TTE: 8.4 × 6.8 × 4.3 cm; CT: 9.6 × 4.5 × 3.8 cm; intra-op: 8.7 × 7.0 cm | Mild mitral regurgitation; left ventricular compression | Surgical resection |
| Tidake A, et al. [125] | 20 | Female | Intermittent fever, chills, headaches → femoral artery embolism | Sinus rhythm | Cardiomegaly, convex left heart border | Yes (TTE) | Yes (MRI) | TTE: 88 × 42 mm | Mild left ventricular hypertrophy; mild pulmonary hypertension; thrombus | Embolectomy for femoral artery thrombus; surgery advised but declined; discharged on warfarin |
| Vagefi PA, et al. [126] | 37 | Male | Asymptomatic | Sinus rhythm, frequent atrial ectopicss, left atrial enlargement | Convexity on left heart border | Yes (TTE) | Yes (CT and MRI) | Intra-op: 11 cm; MRI: 7.2 × 9.3 × 6.0 cm | Mild left ventricular compression | Surgical resection |
| Edyta Płońska-Gościniak, et al. [127] | 25 (initially), 45 (latest follow-up) | Male | Exercise intolerance (NYHA II–III), later palpitations | Paroxysmal atrial flutter and fibrillation (after 15 years) | Cardiomegaly on initial chest X-ray | Yes (TTE and TOE) | Yes (CT and MRI) | Final: 11.2 × 9.0 × 4.8 cm; neck: 3.4 × 2.5 cm | Compression of left ventricle; close relation to left coronary artery | Conservatively managed with β-blockers and acenocoumarol; stable at 20-year follow-up with NYHA II and tolerated arrhythmia |
| Brenneman DJ, et al. [128] | 4 | Female | Asymptomatic | Normal sinus rhythm | Prominence at left mediastinal border | Yes (TTE and TOE) | Yes (CT and MRI) | Not specified numerically | None reported | Surgical resection |
| Ota C, et al. [129] | 1 | Female | Asymptomatic | Sinus rhythm | Bulging upper left heart border without cardiomegaly | Yes (TTE) | Yes (CT) | Intra-op: ~3 cm long, 1–2 cm diameter | Pericardial defect; cardiac levoposition | 5-year follow-up uneventful, then surgical resection |
| Kim YW, et al. [130] | 49 | Male | Palpitations and dizziness | Atrial fibrillation | Not specified | Yes (TTE and TOE) | Yes (CT and MRI) | 57 mm (MRI) | Thrombus | Surgical ablation and AtriClip via right mini-thoracotomy; residual flow on follow-up CT → continued warfarin |
| Wang HQ, et al. [131] | 33 | Female | 2 years of intermittent chest discomfort and one episode of syncope | Sinus rhythm | Cardiomegaly, prominent left atrial appendage | Yes (TTE) | Yes (CT) | TTE: 8.4 × 6.8 cm; Neck: 23 mm | Compression of left ventricle | Surgical resection |
| Asfalou I, et al [132] | 14 | Male | Persistent hiccups for 1 month | Sinus rhythm; left ventricular hypertrophy | Left heart border bulging | Yes (TTE) | Yes (CT) | 69 × 48 mm; neck: 18 mm | None | Surgical resection |
| Bamous M, et al. [133] | 14 | Male | Dry cough for 5 months | Sinus rhythm with left atrial enlargement | Prominent upper left heart border | Yes (TTE) | Yes (CT) | 8 × 6 cm | Deformation of left ventricular lateral wall and pulmonary infundibulum | Surgical aneurysmectomy |
| Toufan M, et al. [134] | 32 | Female | 6-month history of chronic non-productive cough and exertional palpitations | Not specified | Increased cardiothoracic ratio; prominent left heart border | Yes (TTE and TOE) | Not reported | 7.7 × 4.4 cm; neck: 1.0 cm | Left ventricular compression | Surgical resection |
| Hosseini S, et al. [135] | 68 | Female | Atypical chest pain, exertional dyspnea, palpitations | Sinus rhythm with atrial ectopics; transient atrial fibrillation | Cardiomegaly with convex left heart border | Yes (TOE) | Yes (CT) | 5.8 × 4.8 cm; neck: 0.6 cm | Mild to moderate mitral regurgitation; myxomatous mitral valve | Surgical resection + mitral repair |
| Sharma J, et al. [136] | 35 | Female | 2-year history of dyspnea | Sinus rhythm | Prominent mid-left cardiac border; normal heart size | Yes (TTE and TOE) | Yes (CT) | 51 × 66 mm | None | Declined surgery; managed with oral anticoagulation |
| Saygi M, et al. [137] | 35 days | Male | Cyanosis (O2 sat 75%) | Not specified | Not specified | Yes (TTE) | Not performed | 23 × 10 mm | Transposition of great arteries, atrial septal defect, patent ductus arteriosus | Arterial switch, patent ductus arteriosus ligation, downsizing; no intervention on left atrial appendage; |
| Vázquez Antona CA, et al. [138] | 1 | Not specified | Asymptomatic | Not reported | Cardiomegaly | Yes (TTE) | Not performed | 70 mm (length), ostium 22 mm, mid-section 20 mm | Atrial septal defect (fenestrated ostium secundum); dilated right atrial appendage and right heart | Conservative management |
| Li YH, et al. [139] | 57 | Female | Intermittent palpitations | Atrial fibrillation with rapid ventricular response | Bulky mass obliterating the left cardiac border | Yes (TTE) | Yes (CT) | Not specified | None reported | Surgical aneurysmectomy |
| DiBardino DJ, et al. [140] | 6 | Female | Asymptomatic | Normal sinus rhythm | Prominent left heart border | Yes (TTE) | Yes (CT) | Intra-op: 8 × 6 × 4 cm | None | Off-pump lateral thoracotomy with external snare ligation |
| Nakai Y, et al. [141] | 9 | Female | Asymptomatic | Sinus rhythm | Cardiomegaly, bulging left third arch | Yes (TTE) | Yes (CT and MRI) | 67 × 69 × 85 mm | Left ventricular compression | Surgical resection |
| Kapoor S, et al. [142] | 9 | Male | Fever, gross hematuria, anasarca, stroke, seizure, hemiparesis | Tachycardia | Cardiomegaly, elevated left main bronchus | Yes (TTE) | Yes (MRI) | 5.2 × 5.6 cm | Mobile thrombus in left atrial appendage; LV dysfunction (EF 35%) | Surgical resection |
| Shih YJ, et al. [143] | 57 | Female | Abdominal pain, vomiting, cold sweats | Paroxysmal atrial fibrillation | Bulge on left cardiac border | Yes (TOE) | Yes (CT) | TTE: 6.4 × 6.5 cm; intra-op: ~7 × 7 cm; CT: >8.5 cm | None reported | Surgical resection + Cox maze III |
| Kawata M, et al. [144] | 28 | Female | Progressive dyspnea, chest pain | Atrial fibrillation | Cardiomegaly, prominent left border | Yes (TTE) | Yes (CT) | 58 × 92 mm | Severe mitral regurgitation due to annular deformity | Surgical resection + mitral annuloplasty + left-sided Maze procedure |
| Atchison FW, et al. [145] | 45 | Male | Palpitations | Supraventricular tachycardia | Not specified | Yes (TTE and TOE) | Yes (MRI) | TOE: 6.5 × 6.2 cm | Mass effect on left ventricle | Surgical resection + CryoMaze procedure |
| Itaya H, et al. [146] | 63 | Female | Ischemic stroke, left hemiparesis | Chronic atrial fibrillation | Cardiomegaly, enlarged left atrial appendage silhouette | Yes (TTE) | Yes (CT) | 71 × 49 × 42 mm (intra-op) | Tricuspid regurgitation, right atrial enlargement, pulmonary hypertension; thrombus | Surgical resection + full maze + tricuspid annuloplasty |
| Nagai T, et al. [147] | 29 (diagnosed at 19) | Female | Palpitations during exercise | Atrial tachycardia with 2:1 ventricular response (onset at 29 years) | Abnormal upper left heart border | Yes (TTE and TOE) | (Yes (CT) | 66 mm from neck to AT focus; volume: 74–75 mL | None reported | Resection via thoracotomy |
| DeSena HC, et al. [148] | 49 | Male | Palpitations | Atrial fibrillation | Cardiomegaly, left atrial and left ventricular enlargement | Yes (TTE) | Yes (MRI) | 10.5 × 6.5 × 2 cm (intra-op) | Mitral valve dysplasia, severe mitral regurgitation, right ventricular outflow tract obstruction | Surgical mitral valve replacement + Cox-Maze III + left atrial appendage aneurysm resection |
| Wilson D, et al. [149] | 38 | Female | 1-month history of dry cough | Sinus rhythm | Prominent upper left heart border | Yes (TTE) | Yes (MRI) | 9 × 7 cm; neck: 1.7 cm | None | Surgical resection |
| Cho MJ, et al. [150] | 0 (diagnosed prenatally) | Female | Postnatal mild respiratory distress | Not specified | Large protruding left heart border | Yes (foetal echo and post batal TTE) | Yes (CT) | 36 mm (postnatal echo); 22×11 mm (fetal echo) | Moderate-to-severe mitral regurgitation due to left ventricular compression; no valve abnormality | Aneurysmectomy |
| Gupta S, et al. [151] | 2 | Male | Seizure and left hemiparesis; (cerebral infarct) | Sinus rhythm | Cardiomegaly, prominent left heart border | Yes (TTE) | Yes (MRI) | 5.2 × 5.6 cm | Large mobile thrombus | Urgent surgical resection |
| Chowdhury UK, et al. [152] | 28 | Female | Right-sided hemiplegia | Sinus rhythm, left atrial enlargement alternating with recurrent supraventricular arrhythmias, | Massive cardiomegaly, cardio-thoracic ratio 0.75 | Yes (TTE and TOE) | Yes (MRI and CT) | 6 × 5 × 4 cm (echo); 40 × 80 mm (CT) | Moderate left ventricular dysfunction, thrombus in left atrial appendage | Surgical aneurysmectomy |
| Veiga VC, et al. [153] | 28 | Male | 2-year history of palpitations, worsening; | Atrial flutter | Left heart silhouette bulge | Yes (TTE and TOE) | Not done | 7.0 cm (longitudinal axis) | None | Surgical resection |
| de la Fuente A, et al. [154] | 24 | Male | Palpitations | Atrial fibrillation | Enlarged left heart border | Yes (TTE and TOE) | No CT/MRI mentioned | 12.3 × 5.8 cm; neck 1.5 cm | Compressed left ventricle | Surgical aneurysmectomy |
| Selvaraj T, et al. [155] | 28 | Female | Dyspnea, chest pain, palpitations, hemiplegia, aphasia | Atrial flutter | Cardiomegaly with left basal convexity | Yes (TTe and TOE) | CT not mentioned | 8 × 8 × 5 cm | Thrombi in left atrial appendage and left ventricle | Surgical resection |
| Munárriz A, et al. [156] | 24 | Male | Asymptomatic | Atrial fibrillation with slow ventricular response | Prominent left auricular arch | Yes (TTE and TOE) | No CT/MRI performed | TTE: 123 × 58 mm; TOE: 117 × 53 mm; neck: 23–25 mm | Mild mitral regurgitation from annular dilation | Surgical resection |
| Soleimani A, et al. [157] | 27 | Male | 8-month history of palpitations and chest discomfort | Supraventricular tachycardia | Prominent left heart border | Yes (TTE) | No CT/MRI | 9.5 × 9.4 cm; neck: 3 cm | Mild-to-moderate mitral regurgitation | Surgical resection; mitral valve repair |
| Baburaj AK, et al. [158] | 28 | Female | Palpitations | Not specified | Cardiomegaly with clear lung fields | Yes (TTE) | Yes (MRI) | Echo: 10 × 8 cm; Intra-op: 14 × 10 × 8 cm | None | Surgical resection |
| Kühn A, et al. [159] | 2 | Male | Not reported | Atrial flutter with 1:1 AV conduction; ventricular fibrillation during transport | Marked prominence of left upper cardiac border | Yes (TTE and TOE) | Yes (CT) | Not specifically reported | History of large secundum atrial septal defect closed at 1 year | Surgical resection |
| Mathur A, et al. [160] | 60 | Female | Heart failure | Initially atrial fibrillation/flutter; later (post-op) sinus rhythm with 1° atrio-ventricular block, left anterior fascicular block and right bundle branch block | Cardiomegaly | Yes (TTE and TOE) | No CT/MRI reported | 7 × 5 × 3.5 cm | Bi-atrial enlargement | Surgical resection + Cox-Maze |
| León de la Torre RS. [161] | 15 | Female | Sudden-onset stroke (apoplegia) | Atrial fibrillation | Cardiomegaly suggesting dilated cardiomyopathy | Yes (TTE) | No CT/MRI mentioned | Not specified | Mild mitral stenosis, thrombus | Surgical resection |
| Tanoue Y, et al. [162] | 1 | Male | Asymptomatic | Normal | Prominent left cardiac border | Yes (TTE) | Not done | 27 × 19 mm (echo); 29 × 22 mm (surgical) | None | Surgical aneurysmectomy |
| Acartürk E, et al. [163] | 40 | Male | Recurrent embolic strokes (seizure, hemiparesis, dysphagia) | Normal | Slightly prominent upper left heart border | Yes (TTE and TOE) | Yes (MRI) | 6.2 × 2.6 cm (TTE), 6 × 3 × 5 cm (MRI) | None | Surgical resection |
| Chockalingam A, et al. [164] | 12 | Male | Effort intolerance | Supraventricular tachycardia | Not specified | Yes (TTE) | Yes (MRI) | Not specified | Not specified | Surgical resection |
| Pomerantzeff PMAet al. [165] | 33 | Female | Palpitations and chest pain for 10 months | Normal ECG; 24-h Holter showed atrial tachyarrhythmias | Prominent left heart border; mild cardiomegaly | Yes (TTE) | Yes (MRI) | 9.5 × 5.5 cm | None reported | Surgical aneurysmectomy |
| Victor S, et al. [166] | 43 | Female | Dyspnea, palpitations, 3 prior cerebrovascular embolic episodes | Atrial fibrillation | Cardiomegaly | Yes (TTE and TOE) | Yes (MRI) | Not specified; described as "large cucumber-like" with 4 cm neck | Clots | Surgical resection |
| Pome’ G, et al. [167] | 38 (symptomatic at 38; diagnosed earlier) | Female | Paroxysmal palpitations, presyncope | Sinus rhythm | Prominent left heart border | Yes (TTE) | Not done | Echo: 6.9 × 5.6 cm; intra-op: 7.5 × 6 cm | None | Surgical resection |
| Wagshal AB, et al. [168] | 61 | Female | Chest pain | Atrial tachycardia | Prominent left heart border | Yes (TOE) | Yes (CT) | 5.5 × 4.3 cm | Mural thrombus in left atrial appendage | Surgical resection |
| Zhao J, et al. [169] | 27 | Male | Paroxysmal palpitations, NYHA class IV heart failure | Supraventricular tachycardia | Convexity at upper left heart border | Yes (TTE) | Not done | 10 × 10 × 6 cm | Left atrial and left ventricular compression | Surgical resection |
| Kwan CM, et al. [170] | 66 | Female | Paroxysmal palpitations (10+ years), exertional dyspnea (4 years) | Paroxysmal atrial fibrillation (on Holter) | Abnormal bulge in left atrial region | Yes (TTE and TOE) | Yes (CT and MRI) | 15 × 8 × 6 cm (intra-op) | Thrombus | Surgical resection |
| Culver DL, et al. [171] | 88 | Male | Fatigue, dyspnoea, peripheral oedema (heart failure) | Atrial fibrillation alternating with sinus rhythm with inferior Q waves, left anterior fascicular block, right bundle branch block | Cardiomegaly, bilateral pleural effusion | Yes (TTE and TOE) | Not done | Not specified | Moderate mitral regurgitation | Conservative management; no immediate surgical intervention reported |
| Ganeshakrishnan KI, et al. [172] | 35 | Female | Progressive exercise intolerance, fatigue | Atrial fibrillation | Cardiomegaly with prominent left heart border | Yes (TTE) | Yes (CT) | 15 × 10 cm (surgical) | Compressed left ventricle | Surgical excision |
| Comess KA, et al. [173] | 18 | Male | Asymptomatic | Sinus rhythm, left atrial abnormality, -30° axis | Unusual contour of left atrial border | Yes (TTE and TOE) | Not done | 6 × 6 cm (intraoperative) | None | Surgical resection |
| Cujec B, et al. [174] | 42 | Male | Palpitations | Atrial flutter with variable atrio-ventricular conduction; -30° axis | Convexity along left heart border | Yes (TTE and TOE) | Not done | 10 × 8 × 3 cm (surgical) | None | Surgical resection |
| LaRarre TR, et al. [175] | 68 | Male | Recurrent palpitations, syncope | Atrial flutter | Left atrial enlargement | Yes (TTE) | Not done | Not specified | Partial anomalous pulmonary venous return from left lung to right atrium | Managed conservatively with warfarin and verapamil |
| Shirazi SH, et al. [176] | 36 | Female | Asymptomatic except intermittent palpitations | Not specified | Not reported | Yes (TTE) | Not done | Not specified | Hypokinesis of anterolateral left ventricular wall due to aneurysmal compression | Surgical resection |
| Scardi S, et al. [177] | 31 | Female | Asymptomatic | Not specified | Marked prominence of upper left heart border | Yes (TTE) | Yes (CT) | Not specified | None mentioned | Managed conservatively |
| Coselli JS, et al. [178] | 28 | Female | 6-year history of paroxysmal palpitations | Atrial fibrillation | Prominent convexity at upper left heart border | Yes (TTE) | Yes (CT) | 10 cm (intraoperative) | Extrinsic compression of left anterior descending artery left ventricle and left atrium; thrombus | Surgical resection |
| Bramlet DA, et al. [179] | 44 (initial diagnosis); 55 (at death) | Male | Dyspnoea; cerebral embolism | Supraventricular arrhythmia, atrial fibrillation; poor R wave progression | Large, rounded mass on left cardiac border | Yes (TTE) | Not done | 8 × 7 cm with 6 cm orifice (autopsy) | None structurally, but significant coronary displacement; thrombus | No surgery; died of cerebral embolism; thrombus confirmed in aneurysm at autopsy |
| de Feyter PJ, et al. [180] | 34 | Male | Palpitations and dizziness for 3 months | Atrial fibrillation | Abnormal left heart contour | Yes (TTE) | Not done | 6 × 6 × 3.5 cm (surgical) | Compression and hypokinesis of left ventricular anterolateral wall | Surgical resection |
| Tanabe T, et al. [181] | 24 | Female | Palpitations since age 18 | Atrial fibrillation | Cardiac enlargement with convexity on left cardiac border | Yes (TTE) | Not done | ~10 × 10 × 12 cm; neck: 5 × 2 cm | Compression of left atrium and left ventricle | Surgical resection |
| Krueger SK, et al. [182] | 5 | Female | Asymptomatic; incidental finding after respiratory infection | Sinus rhythm; broad notched P waves in lead I, biphasic P in V3R | Prominent convexity at upper left heart border | Yes (TTE) | Not done | 7 × 7 × 4 cm (surgical) | Compression and posterior displacement of left ventricle | Surgical excision |
| Hall J (for Dobbs RH). [183] | diagnosed at age ~2 | Male | Seizures, transient hemiplegia, coma, dysphagia, dysarthria | Sinus rhythm | Globular enlargement of left cardiac border | Not reported | Not done | Not specified | None noted | Anticoagulation from 1962–1966; left atrial appendage aneurysm surgically excised in 1966 |
| Godwin TF, et al. [184] | 26 | Female | Recurrent palpitations (triggered by norepinephrine-containing dental anesthetic), chest pain, presyncope | Atrial tachycardia with varying block | Abnormal contour of left heart border | Yes (TTE) | Not done | ~8 × 5 cm (surgical) | None | Surgical resection |
| Parker JO, et al. [185] | 52 (first symptoms at 42) | Female | 10-year history of paroxysmal palpitations | Normal baseline ECG; paroxysmal atrial flutter with 2:1 atrio-ventricular block during hospitalization | Abnormal left heart contour) | Not done | Not done | Larger than left atrium and left ventricle; precise size not stated | None | Surgery refused; medically managed with digitalis and quinidine |
| Shams KA. et al. [186] | 31 | Male | Congestive heart failure symptoms; cardiogenic shock; history of reumathic heart disease, cachexia, dysphagia | Atrial fibrillation | Abnormal heart position (dextro-position) | Yes (TTE) | Yes (CT) | 130 × 150 mm | Rheumatic valve disease, tricuspid regurgitation, severe pulmonary hypertension, right pulmonary artery compression | Death due to brady-asystole cardiac arrest before surgery |
| Ashworth H, et al. [187] | 38 | Female | Severe right chest pain, exertional dyspnea, cachexia, dysphagia | Atrial fibrillation; later converted spontaneously to sinus rhythm | Gross left atrial enlargement to the right; displaced barium esophagus | Not performed (pre-echocardiography era) | Not applicable | Gross enlargement of left auricle with posterior extension | Severe mitral stenosis, slight tricuspid stenosis, vertebral erosion (T5–T9) | Died of progressive cardiac deterioration; necropsy confirmed massive left atrial appendage dilatation, spinal erosion |
| Semans JH, et al. [188] | 5 | Female | Dyspnea, palpitations | Supraventricular arrhythmia | Cardiomegaly | Not applicable | Not applicable | (6 cm х 10 cm) | Dextrocardia | Death |
| Cassidy MA. [189] | 35 | Female | Palpitations | Atrial fibrillation | There was an enormous cardiac shadow, shaped like a rugby football, along with an acute cardio-phrenic angle. | Not applicable | Not applicable | Not reported | None | Death |
| Ingram A, et al. [190] | 32 | Male | Peculiar bruit on the pulmonary area a few days before the death | Not reported | Not reported | Not applicable | Not applicable | Not reported | None | Death |
| Goyal KK, et al. [191] | 4 | Female | 1-year history of paroxysmal palpitations, presyncope, chest discomfort | Supraventricular tachycardia | Cardiomegaly, prominent left upper cardiac border | Yes (TEE) | Yes (CT) | 3 × 5 cm | Surgical closure of atrial septal defect | Medical therapy (beta blocker, digoxin, amiodarone); asymptomatic; surgery declined by parents |
| Mirmohammadsadeghi M, et al. [192] | 30 | Female | easy fatigability | Sinus rhythm | Abnormal left atrial contour/enlargement | Yes (TEE) | Yes (CT) | ~5 × 4 × 4 cm (intraoperative) | None | Surgical resection |
|  | 46 | Male | Dyspnea on exertion (NYHA III), stroke 2 months prior | Left atrial abnormality | Abnormal left atrial contour/enlargement | Yes (TTE) | Not specified | ~5 × 4 × 4 cm (intraoperative) | History of stroke and dilated coronary sinus suggesting increased atrial pressure | Surgical resection |
| Killinger G, et al. [193] | 32 | Male | Palpitations during sport activity | Atrial fibrillation | Prominent left heart border | Yes (TTE and TOE) | Yes (CT and MRI) | 4.16 × 8.95 cm | None reported | Surgical resection |
| Bolt L, et al. [194] | 86 | Female | Acute anemia, suspected gastro-intestinal bleeding | Persistent atrial fibrillation | Not specified | Yes (TTE) | Yes (CT) | 80 × 46 × 25 mm | Mild–moderate mitral regurgitation; thrombus | Conservative treatment; switched from rivaroxaban to phenprocoumon; patient died 4 weeks later (likely cardiac cause) |
| Sawalha W, et al. [195] | 21 | Male | Palpitations and exertional dyspnea | Atrial fibrillation, | Cardiothoracic ratio 70%, prominent left upper border | Yes (TTE and TOE) | None | 16.5 × 10.5 × 11.8 cm (surgical), histopath: 17 × 12 cm | Distorted left ventricle; displaced left anterior descending artery | Surgical excision |
| Akgün T, et al. [196] | 42 | Female | Exertional dyspnea, palpitations | Sinus rhythm | Mild cardiomegaly | Yes (TTE and TOE) | Not performed | 7 × 5 cm | None | Surgical resection |
| Momtahen M, et al. [197] | 21 | Female | Recent-onset palpitations, exertional dyspnea (NYHA II) | Sinus rhythm with nonspecific ST-T changes | Abnormal upper left heart border | Yes (TTE and TOE) | Yes (CT) | 7.5 × 8.5 × 7.7 cm | Moderate mitral regurgitation, left ventricular systolic dysfunction due to left anterior descending artery compression | Surgical excision |
| Hammad AM, et al. [198] | 19 | Female | Palpitations, dizziness, exercise intolerance | Atrial fibrillation | Prominent left cardiac border | Yes (TTE and TOE) | Yes (MRI) | ~4.5 cm neck; total size not clearly specified, large | Compression of obtuse margin of heart, distorted left anterior descending artery course | Surgical resection |
| Burke RP, et al. [199] | 37 | Male | Syncope at 10, embolic stroke at 14, asymptomatic afterward | Atrial fibrillation, left ventricular hypertrophy | Left heart border enlargement | Yes (TTE) | Not done | 9 × 6 × 8.5 cm | No | Resection and patch repair |
|  | 29 | Male | 6-month history of dyspnea and chest pain | Normal sinus rhythm, T-wave inversion | Left heart border enlargement | Yes (TTE) | Yes (MRI) | 4 × 6 cm | Left anterior descending artery compression and right ventricular outflow tract compression | Resection with stapler |
| Park JS, et al. [200] | 42 | Female | Asymptomatic | Normal sinus rhythm | Progressive cardiomegaly over 18 years | Yes (TTE and TOE) | Not done | 8 × 15 cm | Compression and displacement of left ventricle | Surgical resection |
| Salih AF, et al. [201] | 14 | Male | Palpitations, dizziness | Supraventricular tachycardia documented twice, otherwise normal ECG | Incurvation of left cardiac silhouette | Yes (TTE and TOE) | Yes (CT) | 8.5 × 5.5 cm | Compression of left anterior descending artery (intraoperative finding) | Surgical resection |
| Shati AA, et al. [202] | 2 months | Male | Severe respiratory distress, cyanosis, pneumonia, heart failure | Sinus rm | Large lobulated mass on left cardiac border; left lung collapse | (Yes TTE) | Yes (CT) | 7.2 × 4.2 × 4.3 cm | Severe mitral regurgitation, left lung collapse, mild hypoplasia of left pulmonary artery | Surgical resection |
| Zhang X, et al. [203] | 39 months | Male | Asymptomatic | Sinus rhythm | Enlarged heart border | Yes (TOE) | Yes (MRI) | ~4.5 × 3.2 × 4.9 cm | Mild diastolic dysfunction from left ventricular compression | Surgical resection |
|  | 14 months | Female | Palpitations | Atrial tachyarrhythmia | Not specified | Yes (TTE) | Not performed | 2.9 × 1.0 cm (reduced to 1.2 × 0.7 cm after meds) | Mild mitral regurgitation | Managed with digoxin and amiodarone; heart rate controlled; left atrial appendage aneurysm reduced in size |
|  | 4 months | Female | Asymptomatic | Sinus rhythm | Enlarged heart shadow | Yes (TTE) | Not done | 3.8 × 2.2 cm | Moderate mitral valve regurgitation, left ventricular dilation | No intervention as parents refused further work-up |
|  | 4 years and 4 months | Female | Asymptomatic | Normal sinus rhythm | Enlarged left heart border | Yes (TTE) | Not done | 3.8 × 1.8 cm | Diastolic left ventricular dysfunction | No intervention; lost to follow-up |
|  | 9 years and 1 month | Female | Murmur post-mitral valve valvuloplasty | Sinus rhythm | Not specified | Yes (TTE) | Yes (CT) | 3.3 × 1.9 cm | Moderate mistral stenosis and regurgitation in the setting of prior mitral valvuloplasty | Referred back to original hospital for surgery |
| Ashtekar A, et al. [204] | 20 | Female | Fever, palpitations, exertional dyspnea, chest pain, headache, leg pain | Not specified | Convex opacity along left heart border; peripheral curvilinear calcification | Yes (TTE) | YS (CT and MRI) | Not specified | Thrombus | No specific surgical outcome mentioned; focus was diagnostic imaging |
| Hossain MA, et al. [205] | 21 | Male | Asymptomatic | Not reported | Suspicious for posterior mediastinal mass | Yes (TTE) | Yes (CT) | 90 × 75 mm | None identified | Surgical resection |
| Chung JW, et al [206] | 43 | Male | Palpitations | Atrial fibrillation | Not specified | Yes (TTE) | Yes (MRI) | 34.75 mm (systole), 24.01 mm (diastole) → increased to 46.34 mm and 42.76 mm respectively | None reported | Successful catheter ablation; sinus rhythm maintained for 1 year; left atrial appendage aneurysm enlarged post-ablation with reduced contractility; continued anticoagulation and surgical resection planned |
| Karatasakis GT, et al. [207] | 57 | Female | Palpitations then stroke | Incomplete right bundle block, supraventricular ectopic beats | Prominent middle left cardiac border, cardiomegaly | Yes (TTE and TOE) | (Yes (CT) | 10 × 3 cm (intraoperative) | Thrombus | Surgical resection |
| Bharath G, et al. [208] | 20 | Female | Intermittent palpitations | Atrial fibrillation | Cardiomegaly and prominent left heart border | Yes (TTE) | Yes (CT) | 10.2 × 8.3 × 11.0 cm | None reported | Surgical resection with bovine pericardium patch |
| Renner U et al. [209] | Not specified | Not specified | Gradual onset of atrial fibrillation; progressive left atrial enlargement | Atrial fibrillation | Likely showed cardiomegaly (not explicitly stated) | TTE suggestive of LA aneurysm and possible pericardial defect | MRI, CT, and angiogram suggested pericardial defect; not confirmed | Not provided | Initially suspected pericardial defect; ruled out during surgery | Surgical aneurysmectomy performed; pericardium intact; clinical improvement expected |
| Suciu H, et ak [210] | 6 | Female | Asymptomatic | Sinus rhythm with negative T waves in the precordial leads | Cardiac enlargement (suspected tumour) | Yes (TTE) | Yes (CT) | 6 x 4 cm | Secundum atrial septal defect | Surgical excision of aneurysm |
| Riordan G, et al [211] | 12 | Male | Cough | Not reported | Cardiomegaly | Yes (TTE and TOE) | Yes (CT) | 10x9.1x8.9 cm | Clot | Conservative management |
| Nosáľ M, et al [212] | 4 days | Male | Respiratory  distress | Not reported | Prominence of left atrial border | Yes (TTE) | Yes (CT) | 50 × 25 × 30 mm | PDA, moderate-to-severe mitral regurgitation | Surgical resection |
